# Clinical Evaluation of Melorheostosis in the Context of a Natural History Clinical Study

**DOI:** 10.1002/jbm4.10214

**Published:** 2019-07-26

**Authors:** Smita Jha, Edward W Cowen, Tanya J Lehky, Katharine Alter, Lauren Flynn, James C Reynolds, Eileen Lange, James D Katz, Joan C Marini, Richard M Siegel, Timothy Bhattacharyya

**Affiliations:** ^1^ Clinical and Investigative Orthopedics Surgery Unit National Institute of Arthritis and Musculoskeletal and Skin Diseases (NIAMS), NIH Bethesda MD USA; ^2^ Section on Congenital Disorders NIH Clinical Center Bethesda MD USA; ^3^ Dermatology Branch National Institute of Arthritis and Musculoskeletal and Skin Diseases (NIAMS), NIH Bethesda MD USA; ^4^ EMG Section, National Institutes of Neurological Disorders and Stroke (NINDS), NIH Bethesda MD USA; ^5^ Functional and Applied Biomechanics Section Rehabilitation Medicine Department, NIH Bethesda MD USA; ^6^ National Institutes of Neurological Disorders and Stroke (NINDS), NIH Bethesda MD USA; ^7^ Nuclear Medicine Division Radiology and Imaging Sciences, NIH Clinical Center Bethesda MD USA; ^8^ Office of the Clinical Director National Institute of Arthritis and Musculoskeletal and Skin Diseases (NIAMS), NIH Bethesda MD USA; ^9^ Section on Heritable Disorders of Bone and Extracellular Matrix National Institute of Child Health and Human Development, NIH Bethesda MD USA; ^10^ Immunoregulation Section, Autoimmunity Branch National Institute of Arthritis and Musculoskeletal and Skin Diseases (NIAMS), NIH Bethesda MD USA

**Keywords:** LERI'S DISEASE, SCLEROTIC, SOMATIC MOSAIC, *MAP2K1*, CANDLE‐WAX DISEASE

## Abstract

Melorheostosis is a rare dysostosis involving cortical bone overgrowth that affects the appendicular skeleton. Patients present with pain, deformities, contractures, range of motion limitation(s), and limb swelling. It has been described in children as well as adults. We recently identified somatic mosaicism for gain‐of‐function mutations in *MAP2K1* in patients with melorheostosis. Despite these advances in genetic understanding, there are no effective therapies or clinical guidelines to help clinicians and patients in disease management. In a study to better characterize the clinical and genetic aspects of the disease, we recruited 30 adults with a radiographic appearance of melorheostosis and corresponding increased uptake on ^18^F‐NaF positron emission tomography (PET)/CT. Patients underwent physical exam, imaging studies, and laboratory assessment. All patients underwent nerve conduction studies and ultrasound imaging of the nerve in the anatomic distribution of melorheostosis. We found sensory deficits in approximately 77% of patients, with evidence of focal nerve entrapment in five patients. All patients reported pain; 53% of patients had changes in skin overlying the affected bone. No significant laboratory abnormalities were noted. Our findings suggest that patients with melorheostosis may benefit from a multidisciplinary team of dermatologists, neurologists, orthopedic surgeons, pain and palliative care specialists, and physical medicine and rehabilitation specialists. Future studies focused on disease management are needed. © 2019 The Authors. *JBMR Plus* Published by Wiley Periodicals, Inc. on behalf of American Society for Bone and Mineral Research.

## Introduction

**Figure 1:**
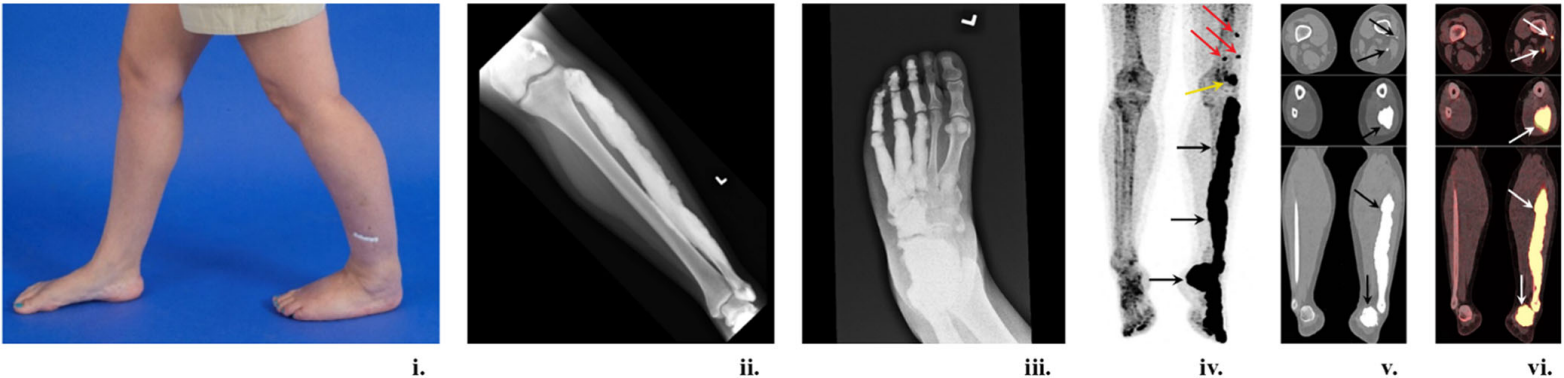
Imaging appearance of melorheostosis. (i) 36‐year‐old woman (Melo‐22) with irregular radial growth of her left leg, (ii) demonstrating classic candle‐wax appearance of the left fibula and lateral three digits on radiograph. (iv) MIP PET ^18^F‐NaF image of her lower extremities showing three small foci of abnormal uptake in the left distal thigh (red arrows), and intensely increased activity in the left lateral femoral condyle (yellow arrow) as well as in the entire left fibula extending to the foot (black arrows). (v, vi) Axial CT and fused ^18^F‐NaF PET/CT images showing ^18^F‐NaF avid focal extraosseous lesions laterally (SUV_max_: 5.32) and posteriorly (SUV_max_: 15.8) to the femur. (v, vi) Axial and coronal CT and fused ^18^F‐NaF PET/CT images showing hyperostosis throughout the left fibula extending to the foot, associated with intensely increased ^18^F‐NaF activity (SUV_max_: 42.5). MIP = maximum intensity projection.

Melorheostosis is a rare hyperostotic bone disease with an estimated prevalence of 1 per million.^1^ Patients commonly present with pain, deformities, limitations of range of motion, contractures, muscle atrophy, and limb swelling Fig 1. Most patients present in childhood or adolescence, with 50% patients being diagnosed by age 20 years.^2^ Germline mutations in *LEMD3* were identified in a patient with melorheostosis and osteopoikilosis.^3^ However, the findings could not be replicated in patients with sporadic melorheostosis.^4^, ^5^ Although first described in 1922, the genetic basis of the disease has only begun to be understood in the past few years. *KRAS* mutation (p.Q61H) was recently identified in the dermatoses of a patient with melorheostosis and familial osteopoikilosis‐ the mutation was not present in the unaffected skin.^6^ The same *KRAS* mutation was also identified in the cervical lymphatic malformation and hyperpigmented skin in a patient with melorheostosis.^7^ Somatic heterozygous activating mutations in *MAP2K1*, resulting in increased proliferation of immature osteoblasts, have been shown to be associated with approximately one‐half of patients with melorheostosis (8/15 patients who underwent paired bone biopsies of affected and unaffected bone), resulting in increased proliferation of immature osteoblasts.^8^ Patients with *MAP2K1*‐positive melorheostosis have a distinct “candle‐wax” appearance on radiographs, characteristic erythematous macular changes on skin overlying melorheostotic bone, and increased unmineralized osteoid on bone histomorphometry.^9^ The underlying pathophysiology of *MAP2K1*‐positive melorheostosis may be explained by gradual deterioration of bone microarchitecture, in turn triggering a periosteal reaction similar to osteomyelitis or trauma, eventually resulting in overall cortical outgrowth.^10^

Melorheostosis has a unique anatomic distribution being limited to appendicular skeleton unilaterally. The diseases progresses proximodistally but limits itself to medial or lateral side of the extremity. This distribution has long been attributed to the distribution of sclerotomes (zone of a skeleton supplied by a single spinal nerve).^11^ However, more recently, findings of CT analysis challenge this hypothesis.^12^ Despite these advances, optimal evaluation or management of the disease is still uncertain, with minimal resources to guide clinicians and lack of a treatment. In this work, we provide our experience with the largest cohort of patients with melorheostosis assembled to date and discuss insights about pathogenesis of symptoms and management of the disease.

## Subjects and Methods

**Figure 2:**
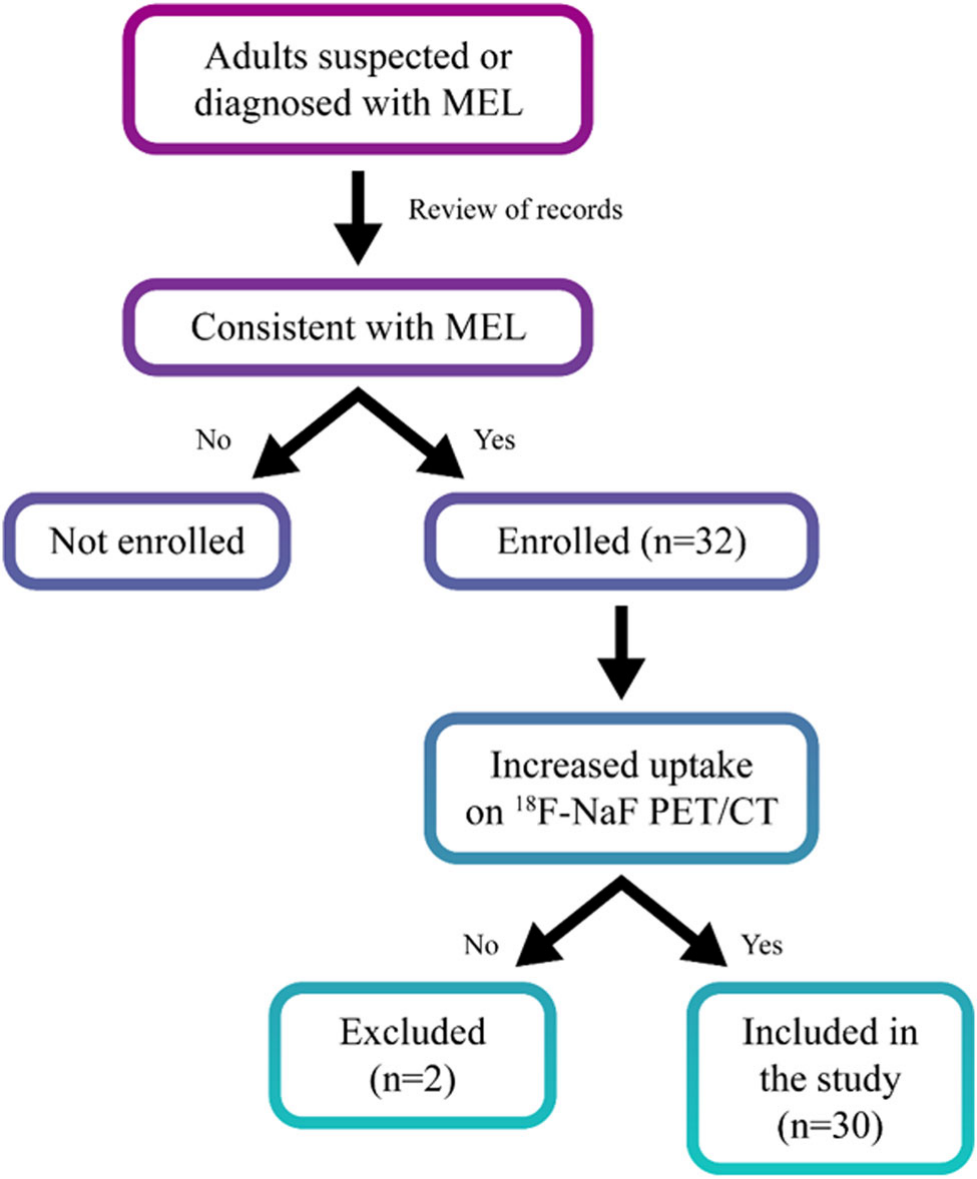
Study design. Flowchart depicts the design for inclusion in the study. All adults with a radiographic appearance of melorheostosis and corresponding increased ^18^F‐NaF uptake were included in the study. MEL = melorheostosis.

The study was approved by the Institutional Review Board of the National Institute of Arthritis and Musculoskeletal and Skin Diseases (NIAMS) (Clinicaltrials.gov NCT02504879) and conducted at the National Institutes of Health (NIH), Bethesda, MD, USA. Patients were eligible to enroll if they were suspected or diagnosed to have melorheostosis and had a radiographic appearance consistent with the disease. Written informed consent was obtained from each patient. We confirmed the diagnosis of melorheostosis by characteristic history, physical examination and X‐rays, and the anatomic correlation of the radiographic abnormality with increased uptake on ^18^F‐NaF (^18^F‐sodium fluoride) positron emission tomography (PET)/CT (Fig. 1). Two patients who were originally enrolled were subsequently excluded. One had abnormal bone thickening associated with a desmoid tumor with relatively mild increase in ^18^F‐NaF uptake. The second was found to have posteromedial tibial stress syndrome with physiologic ^18^F‐NaF activity. Eventually, 30 patients were confirmed to have a diagnosis of melorheostosis (Fig. 2).

All subjects underwent physical exam, imaging studies, and biochemical evaluation of markers of bone turnover. Patients with sensory deficits on neurological exam underwent nerve conduction study (NCS) and ultrasound imaging of the nerves in the anatomic region of distribution of melorheostosis.

Testing included routine blood chemistries, complete blood count, serum calcium, phosphorus, parathyroid hormone intact (iPTH) (electrochemiluminescence immunoassay on Roche Cobas e601 analyzer; Roche Diagnostics, Mannheim, Germany), total vitamin D‐25‐hydroxy (chemiluminescence immunoassay), 1,25 dihydroxyvitamin D (1,25D) (chemiluminescence immunoassay performed on the DiaSorin Liaison XL; DiaSorin, Stillwater, MN, USA), osteocalcin (electrochemiluminescence immunoassay on Roche Cobas e601 analyzer; Roche), alkaline phosphatase (ALP) (Roche Cobas 600 analyzer; Roche), bone‐specific alkaline phosphatase (BSALP) (Mayo Clinic Laboratories, Rochester, MN, USA), type 1 procollagen (β C‐TX) (Mayo Clinic Laboratories; electrochemiluminescence immunoassay on Roche Cobas e601 analyzer; Roche), procollagen I intact N‐terminal (P1NP) (radioimmunoassay; Mayo Clinic Laboratories), urine collagen type 1 cross‐linked N‐telopeptide (NTX) (VITROS competitive chemiluminescence immunoassay; Ortho Clinical Diagnostics, Raritan, NJ, USA), urinary deoxypyridinoline crosslinks (DPD; quantitative enzyme immunoassay, Mayo Laboratories) and 24‐hour urine calcium and creatinine excretion. Additional tests included erythrocyte sedimentation rate (ESR) (Modified Westergren Method), C‐reactive protein (CRP) (Roche Cobas 6000 Analyzer; Roche), lactate dehydrogenase (LDH) (Roche Cobas 6000 analyzer; Roche), creatine kinase (CK) (Roche Cobas 6000 analyzer; Roche), and uric acid (Roche Cobas 6000 analyzer; Roche).

Whole‐body composition dual‐energy X‐ray absorptiometry (DXA) scans were obtained in 10 patients where schedule permitted, with a Hologic Discovery A (Marlborough, MA, USA) densitometer using APEX 4.0 software. Regional area, bone mineral content (BMC), and bone density values (BMD) were compared to data from the National Health and Nutrition Examination Survey (NHANES)^13^ and percentage mean and *Z*‐scores were extracted for both the affected and unaffected contralateral region.

To evaluate the possibility of melorheostosis being caused by a disorder of the nerves, nerve conduction studies were performed using standard methodology on a Nicolet Viking Select Electromyography (EMG) machine (Natus, Middleton, WI, USA) and the results were compared to laboratory‐based normative values. The specific motor and sensory nerves studied were determined by the anatomic distribution of melorheostosis and clinical symptoms. Needle EMG was not performed. Ultrasound scanning was performed using a Phillips Iu22 Ultrasound (Bothell, WA, USA) and included B‐mode and color Doppler scans. A 12‐5 linear transducer was used for lower limb imaging and a 17‐5 linear transducer was used for upper limb studies to identify focal entrapment of large nerves by bone overgrowth. Multiple scans were taken in longitudinal and transverse planes in the region of interest. Both cine‐loops and still images were obtained.

The Patient‐Reported Outcomes Measurement Information System (PROMIS®) is a set of questionnaires to measure different aspects of physical, mental, and social health. PROMIS pain interference (PROMIS‐PI) has been evaluated in individuals with osteoarthritis among other chronic conditions.^14^, ^15^ We used pain interference to assess the consequences of pain on engagement with social, cognitive, emotional, physical, and recreational activities. PROMIS‐PI is based on banks of items calibrated using the graded response model that estimates severity and discrimination (ability to distinguish among people with different levels of pain). The measures were administered via Assessment Center^SM^, a Web‐based data collection platform. PROMIS® item banks have a 7‐day time frame. We used computer adaptive testing (CAT), a tailored approach in which the questions administered were selected based on individuals’ previous responses. PROMIS® uses a *T*‐score metric in which 50 is the mean of a relevant reference population and 10 is the standard deviation (SD) of that population. Scores 0.5 to 1.0 SD above the mean imply mild impairment and scores ≥ 2.0 SD imply severe impairment with scores between 1.0 and 2.0 SD implying moderate impairment.

## Results

Thirty unrelated patients (mean age, 46 years) with melorheostosis of varying sites of disease involvement were studied. The skeletal disease burden ranged from one isolated bone affected to most bones in unilateral or bilateral extremities. Patients described symptoms of melorheostosis for mean duration of 30 years (Table 1). All patients reported pain and limitation of physical function. However, mean *T*‐scores on PROMIS‐PI of patients were notably within the SD of mean of reference population. There was no history of fragility fracture in any patient. No patient reported a family history of melorheostosis or other previously described co‐occurring bone diseases such as osteopoikilosis or Buschke‐Ollendorff Syndrome.

Skin abnormalities overlying the affected bone lesions were identified in 16 of 30 patients (53%). Of 16 patients, 9 (56%) patients’ skin findings manifested as vascular changes, most commonly irregular macular erythema without overlying surface change. Two patients had tan/brown hyperpigmentation of the affected lower extremity, which could represent mild stasis changes or hemosiderin deposition due to impaired venous return. As previously described, histological review of skin samples from patients with *MAP2K1*‐positive melorheostosis showed thickened vascular wall and increased density of superficial vasculature in skin overlying affected bone in comparison to skin from the contralateral extremity.^16^ This pattern was not noted in skin samples from patients with *MAP2K1*‐negative melorheostosis.

**Table 1:** Characteristics of Study Cohort

Patient characteristic	*n* (%)
Gender	
Female	21 (70)
Male	9 (30)
Sidedness	
Right‐sided disease	16 (53)
Left‐sided disease	14 (47)
Upper versus lower extremity	
Lower extremity disease	18 (60)
Upper extremity disease	11 (37)
Diffuse	1 (3)
Age at symptom onset	
≤10 years	11 (37)
11–20 years	11 (37)
21–30 years	4 (13)
31–40 years	3 (10)
51–60 years	1 (3)
Age at study enrollment	
21–30 years	4 (13)
31–40 years	5 (17)
41–50 years	10 (33)
51–60 years	7 (23)
61–70 years	4 (13)

All four extremities affected with melorheostosis.

**Figure 3:**
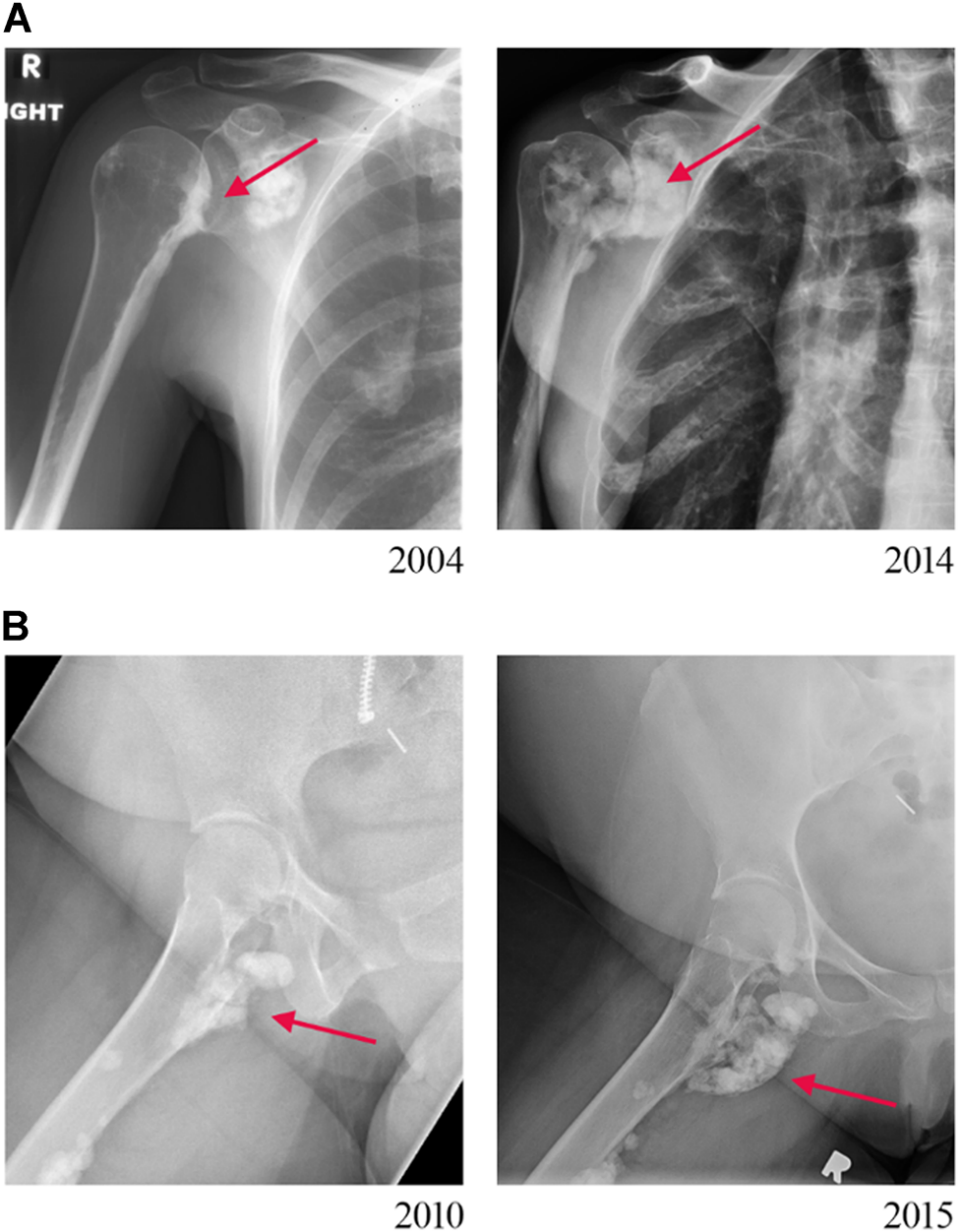
Radiographic progression in melorheostosis. (*A*) Comparison of radiographs from 2004 (left) and 2014 (right) showing progression of both skeletal and extraosseous lesion of melorheostosis (Melo‐21; *MAP2K1* mutation negative). (*B*) Comparison of radiographs from 2010 (left) and 2015 (right) showing evidence of disease progression in the extraosseous lesion around patient's right hip joint (Melo‐6; MAP2K1‐mutation positive).

Sixteen patients (53%) had an osteoma‐like appearance to their lesions on radiographs, whereas 12 (40%) had the classic “dripping candle‐wax” pattern of melorheostosis, with exuberant expansile lesions and thick undulating ridges. Two patients had a mixed radiographic pattern, with a combination of classic and myositis ossificans‐like pattern. There was no difference between patients with osteoma‐like or “dripping” candle‐wax like pattern on radiographs in terms of sclerotomal distribution and spread to axial skeleton. Patients with classic “dripping candle‐wax” appearance on radiographs were, however, more likely to have extraosseous mineralization, commonly periarticular (7/12 patients with classic appearance versus 1/16 patients with “osteoma‐like” appearance). Polyostotic disease was noted in all but two patients. Lesions crossed over joints to affect contiguous bones or skipped a bone to involve other noncontiguous bone(s). We found evidence of axial skeleton involvement in three patients, with melorheostosis extending to the spine and sternum, although even in these patients, the majority of the skeletal burden of disease was in the long bones. Extraosseous lesions were found in 10 patients, seven of which were periarticular. These extraosseous masses were not palpable on exam and were intramuscular. Seven of nine patients showed evidence of disease progression on comparison to historical radiographs (Fig. 3 *A* and *B*).

Whole‐body DXA comparing the affected extremity to the unaffected showed that in patients with significant skeletal disease burden, BMC was markedly elevated whereas the measured area was only marginally elevated accounting for a significant increase in BMD in the affected extremity (Table 2). In patients with low skeletal disease burden, changes in BMD were much less striking. T‐scores at total hip ≤–3 were noted in two patients, whereas historical records revealed an additional patient with the lowest T‐score (–1.6) at femoral neck.

All patients had intact cell counts without anemia, leukopenia, or thrombocytopenia. Mean serum calcium and phosphorus were 2.23 mmol/L (reference range, 2.15 to 2.55 mmol/L) and 3.78 mg/dL (reference range, 2.5 to 4.5 mg/dL), respectively. No clinically meaningful information could be surmised from markers of bone formation (BSAP, osteocalcin, and amino‐terminal pro‐peptide) and resorption (carboxy‐terminal pro‐peptide, NTX, and deoxypyridinoline [DYD]). Erythrocyte sedimentation rate (ESR) and CRP were normal in the majority of patients. No significant abnormalities in serum LDH, uric acid, and CK were noted.

**Table 2:** Whole‐Body DXA

		Affected extremity						Unaffected contralateral extremity					
		Area		BMC		BMD		Area		BMC		BMD	
Study ID	Region	Mean (%)	*Z*‐score	Mean (%)	*Z*‐score	Mean (%)	*Z*‐score	Mean (%)	*Z*‐score	Mean (%)	*Z*‐score	Mean (%)	*Z*‐score
Melo‐04	RLE	91.44	–0.86	85.93	–0.90	94.36	–0.64	103.63	0.37	108.78	0.56	105.41	0.61
Melo‐06	RLE	93.42	–0.68	137.22	2.40	**147.37**	**5.22**	109.63	1.00	106.11	0.39	97.14	–0.31
Melo‐18	LUE	54.98	**–4.16**	163.28	**4.11**	298.10	**26.55**	102.96	0.29	105.44	0.36	102.85	0.38
Melo‐20	RUE	94.63	–0.38	112.82	0.87	119.60	**2.81**	93.57	–0.61	119.44	1.29	128.19	**3.93**
Melo‐21	RUE	97.40	–0.28	96.48	–0.22	99.49	–0.06	97.20	–0.28	100.70	0.04	104.09	0.45
Melo‐22	LLE	111.48	1.20	167.60	**4.40**	150.86	**5.83**	96.27	–0.39	95.47	–0.30	99.54	–0.05
Melo‐26	LLE	86.58	–1.35	91.48	–0.50	106.13	0.61	97.59	–0.24	98.67	–0.08	101.61	0.16
Melo‐27	RUE	69.69	**–3.21**	108.91	0.57	157.06	**6.66**	93.41	–0.68	107.43	0.48	115.46	1.82
Melo‐28	RUE	98.24	–0.17	132.72	2.17	135.67	**4.72**	N/A	N/A	N/A	N/A	N/A	N/A
Melo‐28	LUE	90.08	–0.92	128.41	1.84	143.07	**5.77**	N/A	N/A	N/A	N/A	N/A	N/A
Melo‐29	LLE	85.35	–1.48	100.43	0.03	118.19	1.80	102.76	0.28	101.61	0.10	99.36	–0.06

Whole‐body DXA findings comparing regional area, BMC, and BMD of the extremity affected with melorheostosis to the unaffected contralateral extremity. Findings showed that in patients with significant skeletal disease burden, BMC was markedly elevated whereas area was only marginally elevated accounting for a significant increase in BMD in the extremity affected with melorheostosis in comparison to the unaffected extremity. In patients with low skeletal disease burden, changes in BMD were much less striking. All values are referenced to data from NHANES. Affected regions are represented as RUE, LUE, RLE, and LLE.

DXA =  dual‐energy X‐ray absorptiometry; BMC = bone mineral content; BMD = bone mineral density; RLE = right lower extremity; LUE = left upper extremity; RUE = right upper extremity; LLE = left lower extremity; NHANES = National Health and Nutrition Examination Survey.

**Figure 4:**
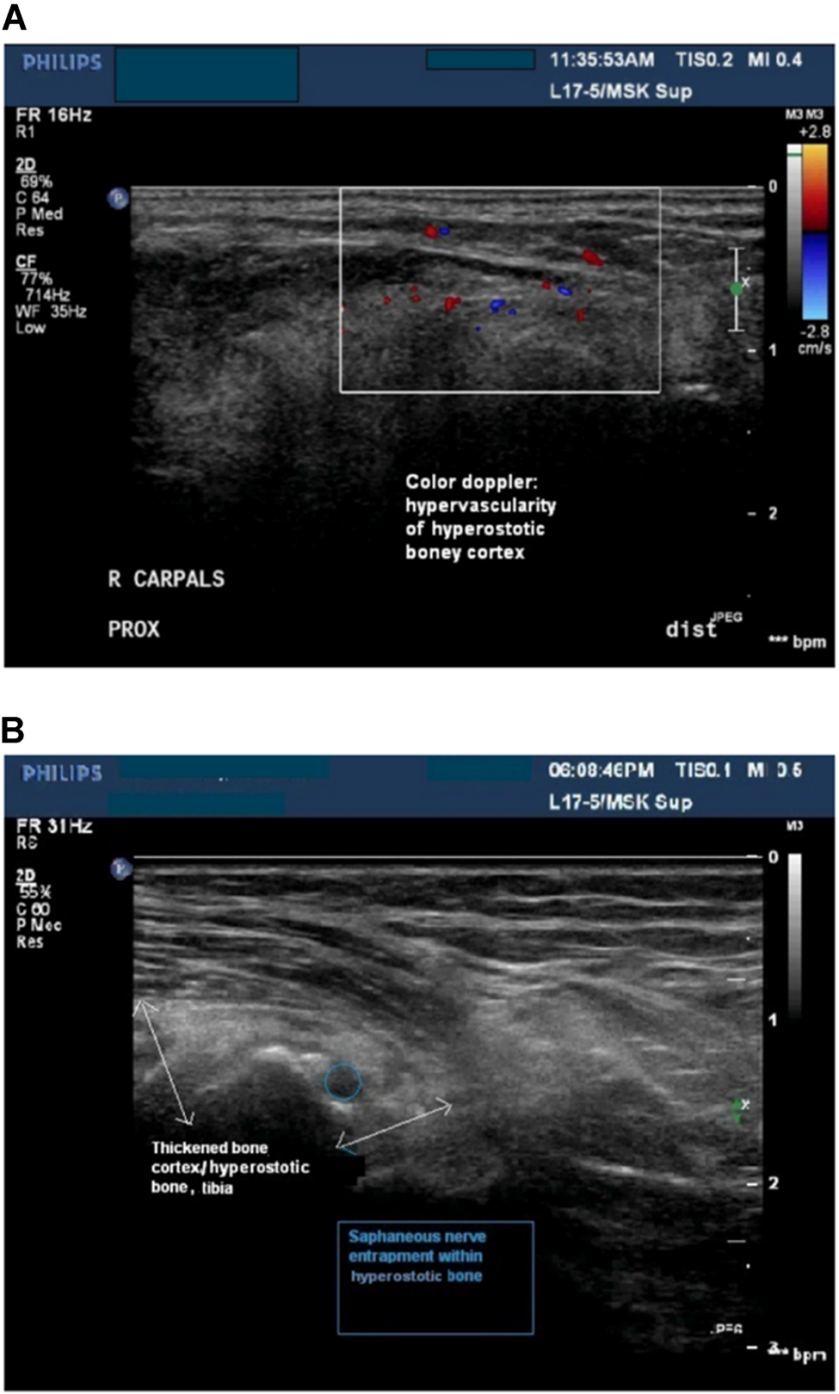
Neurologic consequences of melorheostosis. (*A*) Ultrasonogram imaging showing hypervascularity of the hyperostotic bone cortex (white box). (*B*) Ultrasonogram imaging (Melo‐10) showing hyperostotic bone (white arrows) with focal entrapment of the saphenous nerve (blue circle). The entrapped nerve was the likely cause of patient's pain, numbness, and tingling.

Neurological clinical evaluation showed all 30 patients reporting pain symptomology with 77% patients with confirmed sensory deficit in the distribution of melorheostosis on exam. Motor exam showed restriction of joint movement due to bony growth in 16 patients. There was no focal muscle weakness except that due to restriction of joint mobility related to melorheostosis. Eight patients had muscle atrophy from disuse. Electrophysiological testing identified isolated neuropathy in 43% patients (12/28 patients tested, with six sensory neuropathies and six mixed motor neuropathies) in the distribution of melorheostosis with two other patients having a generalized sensory neuropathy. Ultrasound imaging detected cortical thickening and irregularity of the affected bone(s) in all 25 patients in whom it was performed. Twelve (12) patients (50%) showed marked hypervascularity of the cortex and adjacent soft tissue. One study showed the saphenous nerve encapsulated within the bone overgrowth (Fig. 4 *A* and *B*) and in one patient with isolated sural neuropathy, enlargement of the sural nerve was noted in the distal third of the lateral leg, proximal to a melorheostotic lesion. Three additional ultrasound studies showed either displacement or swelling of a nerve due to compression by the bone overgrowth. There was no significant difference in prevalence of neuropathy between patients with classic “dripping candle‐wax” appearance and osteoma‐like appearance on radiographs (81% in osteoma‐like versus 67% in classic).

## Discussion

In this most comprehensive study of melorheostosis to date, we identified a number of clinical features associated with this hyperostotic bone disease. Melorheostosis is a skeletal dysostosis of cortical bone overgrowth along multiple contiguous bones in one or more extremities. Lesions are characterized by abnormally increased uptake of ^99m^Tc‐MDP on bone scintigraphy and ^18^F‐NaF on ^18^F‐NaF PET/CT imaging, which distinguishes melorheostosis from osteopoikilosis.^17^

PROMIS‐PI scores within the SD of the reference population suggest that the chronic pain commonly experienced by patients with melorheostosis often does not interfere significantly with their daily activities. However, the functional impact of the disease is potentially related to the distribution of the disease, because patients with significant disease in their hand or foot (in contrast to arm and leg) are likely to experience greater occupational disability as a consequence.

Our data illuminates some mechanisms for pain and functional impairment in limbs affected by melorheostosis, highlighting effects on sensory nerves. All patients had pain and a majority had focal sensory deficit on clinical exam in the distribution of melorheostosis. Less than one‐half the patients had focal electrodiagnostic abnormalities, which were all within the area of the bony defects. These focal abnormalities are likely related to displaced or encased nerves such as was observed by ultrasound imaging in five patients. In patients with nondiagnostic nerve conduction and ultrasound imaging, the symptoms may be explained by compression or irritation of *small* sensory nerve endings close to the bony surface. These abnormalities are beyond the sensitivity of nerve conduction and ultrasound. Ultrasound imaging was also useful in detecting hypervascularity in and around the melorheostotic lesions, correlating with the hypervascularity noted on bone histology and further supported by ^18^F‐NaF PET/CT imaging. It is not known if the observed hypervascularity contributes to pain. Last, bony growth and joint deformities that restricted joint mobility were noted in 53% of patients and may have contributed to discomfort.

It is worth noting that melorheostosis—a disease of bone overgrowth can coexist with osteoporosis—a disease of low bone mass. This is consistent with current knowledge that melorheostosis is associated with somatic mosaic mutations in circumscribed areas of skeleton whereas osteoporosis is a complex genetic trait further modified by systemic, environmental, and nutritional factors, which has a generalized distribution. Hence, we believe that patients with melorheostosis should undergo routine screening and management of osteoporosis according to standard guidelines although the risks of antiresorptives such as bisphosphonates or anabolic agents such as teriparatide in melorheostosis are not clear. We did not find utility of whole‐body DXA scans in diagnosis of melorheostosis.

As expected, melorheostosis appears to spare overall hematopoietic marrow as shown by preserved counts on laboratory assessment. Progression of disease demonstrated on serial radiographs suggests that the disease progresses over time—both within a bone and to adjacent bones, occasionally even skipping bones. We speculate that the rate of disease progression varies with time and differs between individuals and also between different lesions in the same patient. It is unclear if periods of disease progression correspond with increase in pain.

Based on our extensive studies of these 30 patients, we recommend the following diagnostic studies for melorheostosis. Clinical history and physical exam should be focused on ascertainment of patient's symptoms, family history, and anatomic distribution of any visible or palpable bone irregularity and associated skin changes. Routine radiographs and ^18^F‐NaF PET/CT scans could be obtained to assess disease burden and monitor disease activity, although it is currently available only at major medical centers. ^18^F‐NaF PET/CT scans are, however, not necessary for diagnosis of melorheostosis. Bone scintigraphy or ^18^F‐NaF PET/CT also assists in exclusion of osteopoikilosis from the differential diagnosis and in detection of distant sites of melorheostosis.^17^, ^18^, ^19^ Classic candle‐wax appearance with exuberant growth of cortical bone seen on radiographs has been shown to be predictive of *MAP2K1*‐positive melorheostosis.^9^

Additional laboratory studies can be beneficial to evaluate the overall skeletal health. However, we did not find any biochemical abnormalities characteristic of the disease. Lack of marked abnormalities in markers of bone turnover deserves further investigation but may be explained by the focal nature of the disease because bone turnover markers reflect the turnover of the entire skeleton and integrate the variations.^20^ Clinical evaluation by a dermatologist not only offers reassurance to patients, but findings in skin overlying the lesion(s) in bone can predict the presence of *MAP2K1*‐positive melorheostosis because disease associated with *MAP2K1* mutations is more often associated with vascular lesions in overlying skin.^9^ Cutaneous lesions suggestive of *KRAS* mutations may also be identified on skin exam. Evaluation by a neurologist can help in identifying a suitable analgesic regimen.

Treatment for melorheostosis remains symptomatic. Our clinical experience suggests that patients with the disease benefit from reassurance about the rarity of malignant transformation of lesions and evaluation by rehabilitation medicine and pain management specialists. Although there are at least three case reports of malignancy in patients with melorheostosis, it is not clear if osteosarcoma arose in the precise distribution of melorheostosis in these three cases described. Patients should be advised to seek medical attention in the context of rapid progression or worsening symptoms. Given the high prevalence of nerve entrapment from melorheostotic bone expansion, drugs such as pregabalin and gabapentin may be considered for management of neuropathic pain. The finding of markedly increased ratio of RANKL/OPG transcripts in osteoblasts from affected bone in patients with *MAP2K1*‐positive melorheostosis raises the possibility of using RANKL inhibitors in these patients,^21^ although it is possible that the increased ratio of RANKL/OPG transcripts is actually a compensatory mechanism for the dense bone. Case reports describing the use of bisphosphonates resulting in symptomatic and scintigraphic improvement of melorheostosis have also been published.^22^ Given accelerated bone remodeling in affected bone of patients with *MAP2K1*‐positive melorheostosis, it is biologically appealing to postulate a role for antiresorptives. However, the clinical benefit with antiresorptives is counterintuitive given the enhanced osteoblast growth seen in *MAP2K1*‐positive melorheostosis. Interestingly, coupling between osteoblasts and osteoclasts is retained.^8^ Nevertheless, melorheostosis is a focal disease, which affects a small part of the skeleton in most patients, and we believe that bisphosphonates should not be withheld in patients with both melorheostosis and osteoporosis. Controlled clinical trials to study the effect of bisphosphonates on melorheostotic lesions is warranted. Symptomatic improvement of pain and vasomotor function with the use of nifedipine, a vasoconstrictor, has been described in melorheostosis.^23^ In the context of our findings of hypervascularity on histology and ultrasound, this seems plausible for symptomatic management, though it would not alter the progression of disease.^9^

Two patients underwent surgical resection of melorheostotic lesions because of significant limitation of daily activities. In general, we do not recommend surgical intervention because melorheostotic bone is technically difficult to operate on and because the disease can recur.^24^ We have not noted any recurrence of bone growth in 1‐year follow‐up postsurgery in these patients.

Although we did not find any significant difference in prevalence of neuropathy, extraosseous mineralization, or conformation to single or contiguous sclerotome between the groups with classic “dripping candle‐wax” or “osteoma‐like” radiographic appearance, continued experience with more patients will help enhance our understanding of these two patterns of the disease.

*MAP2K1* codes for protein MEK1 protein kinase and the activating mutations we identified in melorheostosis result in increased signaling of the RAS/MAPK pathway. MEK‐inhibitors such as trametinib have been developed for patients with cancer but their use is currently limited due to toxicities. Future studies on targeted treatment and development of MEK inhibitors with better therapeutic index may allow targeted therapy in patients with *MAP2K1*‐positive disease.

## Disclosures

All authors state that they have no conflicts of interest.
